# The 1,10-Phenanthroline Ligand Enhances the Antiproliferative Activity of DNA-Intercalating Thiourea-Pd(II) and -Pt(II) Complexes Against Cisplatin-Sensitive and -Resistant Human Ovarian Cancer Cell Lines

**DOI:** 10.3390/ijms20246122

**Published:** 2019-12-04

**Authors:** Gaetano Marverti, Gaia Gozzi, Angela Lauriola, Glauco Ponterini, Silvia Belluti, Carol Imbriano, Maria Paola Costi, Domenico D’Arca

**Affiliations:** 1 Department of Biomedical, Metabolic and Neural Sciences, University of Modena and Reggio Emilia, Via Campi 287, 41125 Modena, Italy; g.gozzi@holostem.com (G.G.); angela.lauriola@univr.it (A.L.); 2 Department of Life Sciences, University of Modena and Reggio Emilia, Via Campi 213/d, 41125 Modena, Italy; glauco.ponterini@unimore.it (G.P.); silvia.belluti@unimore.it (S.B.); carol.imbriano@unimore.it (C.I.); mariapaola.costi@unimore.it (M.P.C.)

**Keywords:** cisplatin-resistance, DNA intercalators, ovarian cancer cells, topoisomerase II, thymidylate synthase, bipyridine, phenanthroline, Pt(II), Pd(II), thioureas

## Abstract

Ovarian cancer is the most lethal gynecological malignancy, often because of the frequent insurgence of chemoresistance to the drugs currently used. Thus, new therapeutical agents are needed. We tested the toxicity of 16 new DNA-intercalating agents to cisplatin (cDDP)-sensitive human ovarian carcinoma cell lines and their resistant counterparts. The compounds were the complexes of Pt(II) or Pd(II) with bipyridyl (bipy) and phenanthrolyl (phen) and with four different thiourea ancillary ligands. Within each of the four series of complexes characterized by the same thiourea ligand, the Pd(phen) drugs invariably showed the highest anti-proliferative efficacy. This paralleled both a higher intracellular drug accumulation and a more efficient DNA intercalation than all the other metal-bidentate ligand combinations. The consequent inhibition of topoisomerase II activity led to the greatest inhibition of DNA metabolism, evidenced by the inhibition of the expression of the folate cycle enzymes and a marked perturbation of cell-cycle distribution in both cell lines. These findings indicate that the particular interaction of Pd(II) with phenanthroline confers the best pharmacokinetic and pharmacodynamic properties that make this class of DNA intercalators remarkable inhibitors, even of the resistant cell growth.

## 1. Introduction

The importance of intercalating drugs in the treatment of solid tumours, including ovarian cancer, has been highlighted by a recent guidance from the National Institute for Health and Care Excellence (NICE) that recommends pegylated liposomal doxorubicin hydrochloride (PLDH), alone or alongside platinum, for treating recurrent ovarian cancer [1].

Many of the clinically used DNA-intercalating agents are potent inhibitors of both DNA and RNA synthesis [2,3], and this is considered their primary mode of action. In addition, DNA damage, for example, through micronuclei formation, can result from interference of most DNA-intercalating agents with topoisomerase II (TOPO-II), an enzymatic protein with the role of maintaining the correct topological properties of DNA in cells [3].

As a development of studies on non-covalent interactions of square-planar Pt(II) complexes intercalating the DNA duplex [4], bipyridyl (bipy) complexes of Pt(II) with thiourea were reported as a new class of efficient DNA intercalators that exploit (i) the bipy moiety to insert between base pairs, (ii) the Pt(II) core size and coordination to impose molecular geometry and provide the positive charge, and (iii) the strong binding of the thiourea ligands to Pt(II) to avoid unwanted covalent reactions with nucleobases [5].

The biological activities of some bipy complexes of Pt(II) with various substituted thioureas, designed to intercalate into DNA, were tested against cisplatin (cDDP)-sensitive and –resistant human ovarian carcinoma cell lines [6]. cDDP is an anticancer drug in clinical use for the treatment of malignancies of the urogenital tract and other solid cancers [7]. However, its clinical success is often diminished by intrinsic and acquired tumour resistance. This is a multifactorial process that also includes specific cellular modifications that have been shown to be responsible for resistance to DNA-intercalating agents or to be present in cell lines, which display cross-resistance to both classes of drugs. In this regard, cross-resistance to the DNA intercalator doxorubicin was observed in cDDP-resistant cancer cell lines with high levels of glutathione, and/or an increased DNA TOPO-II activity and with a P-gp-mediated multi-drug resistance phenotype [8].

We have shown that the anti-proliferative efficacy of tested Pt(II)-bipyridyl-substituted thiourea complexes increases with the ancillary thiourea-ligand bulkiness and the hydrophobicity of its substituents. In particular, the presence of two phenyl groups on the thiourea moiety confers them an outstanding cytotoxicity, even against cDDP-resistant cells. Cell growth inhibition partially paralleled drug accumulation. Instead, drug intercalation into DNA was favoured by the planarity of the bipyridyl mojety [9].

Subsequently, the chemical variety of these complexes was significantly widened by the synthesis and structural characterization of several Pd(II) bipy and 1,10-phenanthroline (phen) complexes with *N*-alkyl substituted thioureas as the ancillary ligands [10] and Pt(II) [11]. The capability of 1,10-phenantroline to interact with nucleic acid base pairs has been clearly explained [12]. Both AT and, more strongly, GC base pairs bind phenanthroline. The predicted stacked structure of the complex of phen with AT base pairs is found complementary to the available crystal structure. However, in certain crystal structures, the phen ligand is found partially intercalated within two thymine nucleobases [13].

Recently, Barra et al. [14] have addressed the structural determinants of the bioactivity of Pd(II) complexes of general formulae [Pd(phen)(tu)_2_]^2+^, incorporating the intercalator phen and thiourea (tu) with the latter either unsubstituted or *N*-methyl- or *N*,*N*-dimethyl-substituted. DNA binding experiments suggested that the ancillary thiourea ligands did not affect the intercalating ability, as already observed for Pt-bipy-thiourea complexes [6]. These observations indicate that the DNA binding affinity of these compounds is only a partial determinant of the observed cytotoxicity differences. Indeed, pharmacological targets other than TOPO-II cannot be excluded. In this light, a study on complexes of the type [Pt(N–N)(L–L)]^2+^ (N–N = phen derivatives, L–L = bidentate ancillary ligand) highlighted the influence of the ancillary ligand, particularly in terms of interactions with non-DNA receptors [15].

**Figure 1:**
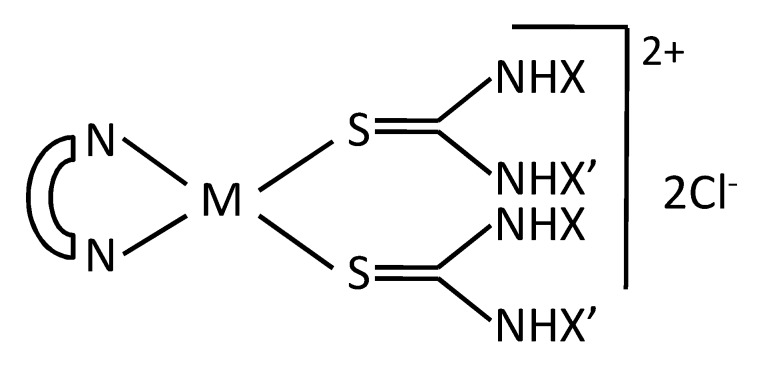
General chemical structure of Pt(II) and Pd(II) (M) complexes with 1,10-phenanthroline or 2,2′-bipyridine (represented as the bidentate ligand, 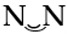) and thioureas. A: X = X′ = H; B: X = H, X′ = CH3; C: X = H; X′ = *n*-Bu; D: X = X′ = C2H5. The Series A, B, C, and D include the complexes with the indicated substituents on thioureas.

In the present work, we tested the relevance of the nature of the metal ions, Pt(II) and Pd(II), as well as of the bidentate (bipy and phen) and ancillary ligands, on the cytotoxicity of metal-bidentate ligand-thiourea complexes in ovarian cancer cells, also shedding light on such mechanistic determinants as cellular internalization of the complexes and their ability to intercalate DNA and to inhibit TOPO-II. We evaluated and compared biological and some correlated biochemical activities of four series of compounds, each characterized by a different thiourea ligand, namely, unsubstituted, methyl-, *n*-butyl-, and di-ethyl-substituted thiourea. Each series included the four complexes of Pt(II) and Pd(II) with bipy and phen and the thiourea (see Figure 1). The sixteen combined complexes were characterized for their ability to intercalate DNA and tested in cDDP-sensitive human ovarian cancer cell lines and in their resistant counterparts, whose resistant phenotype was accompanied by some of the intracellular modifications conferring resistance to DNA-intercalating agents.

## 2. Results

### 2.1. Comparison of the Cytotoxic Efficacy of the Different Complexes

To highlight the role of the substituents on the thiourea moiety, the Pt(phen) and Pd(bipy) complexes were divided into four series: Series A, no substituents; Series B, methyl; Series C, *n*-buthyl; and Series D, bis-ethyl (Figure 1).

**Figure 2:**
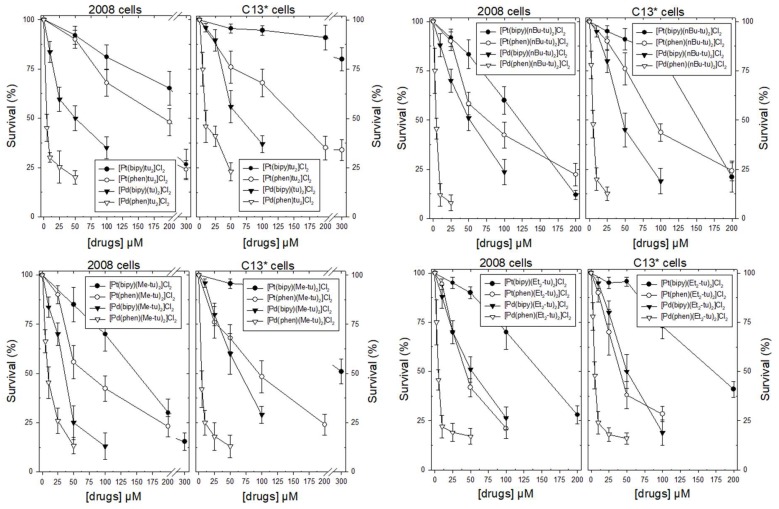
Dose-dependent effects of Pt- and Pd-thiourea complexes of the A and B series (left panels) and of the C and D series (right panels) on the growth of 2008 and C13* cells after a 2 day exposure. Results represent the mean of three separate experiments performed in duplicate. Error bars, standard error of the mean (SEM).

Cytotoxic activity of the complexes of the four series was studied against two pairs of human ovarian carcinoma cell lines, the cDDP-sensivitive 2008 and A2780, and their resistant counterparts, C13* and A2780/CP (Figure 2 and Table S1). As shown in Figure 2, the order of increasing potency on all the cell lines was Pt(bipy) < Pt(phen) < Pd(bipy) < Pd(phen). In particular, the Pt(bipy) complexes were the least toxic, with IC_50_ values ranging from about 120 to 240 µM on sensitive cell lines, and from 150 to 445 µM on resistant cell lines (Table S1). On the other hand, the Pd(phen) complexes were the most active ones against all cancer cell lines, displaying low µM IC_50_ values (Table S1). The Pt(phen) and Pd(bipy) complexes showed similar dose-response curves as well as IC_50_ values.

Interestingly, among the Pt(II)-thiourea complexes with either bipy or phen, the cytotoxicity was increased by the presence of binding ancillary ligands with respect to unsubstituted thiourea complexes, confirming a previous report [6]. This effect occurred to a lesser extent with Pd(II)-thiourea complexes. Nevertheless, the Pd(phen) drugs showed the highest effectiveness whether or not they bore ligands on the respective thioureas.

Their cytotoxic potency was also compared to both cDDP and doxorubin (Table S2), resulting in actually being lower than doxorubicin; however, in the resistant cells they were more or equally effective to cisplatin. As a result, the related resistant factor (RF) values were better than those obtained with both chemotherapy drugs, even with doxorubicin, indicating that the Pd(phen) complexes were able to kill cancer cells even in the presence of the resistance phenotype.

### 2.2. Remarkable Intracellular Accumulation of the Pd(phen) Complexes

Looking for a correlation between the cytotoxicity and the cellular uptake of the complexes, we determined the ^195^Pt and ^105^Pd intracellular concentrations from total cell homogenates using inductively-coupled plasma mass spectrometry (ICP-MS), a technique characterized by very low detection limits for most elements, 1 to 10 ppt for Pd and <0.1 to 1 ppt for Pt.

**Figure 3:**
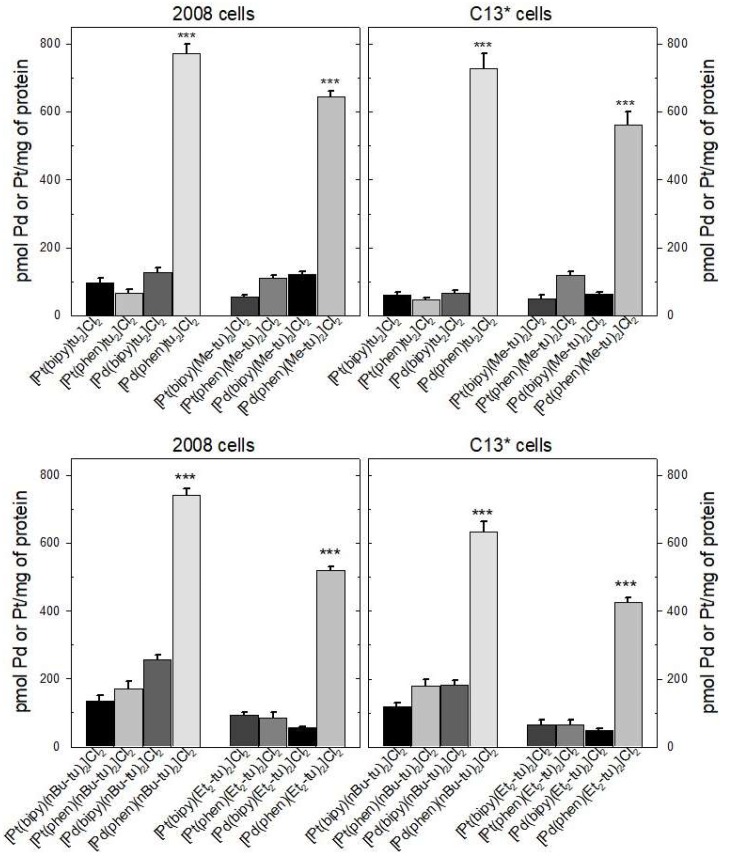
Comparison of Pt and Pd accumulation in 2008 and C13* cells 1 day after exposure to 5 µM of the indicated complexes. The results represent the mean of three experiments conducted with duplicate plates. Error bars, SEM. *** *p* < 0.001 when comparing the Pd(phen)s with the other complexes.

To better compare the accumulations of very active complexes with those of the scantily cytotoxic forms, 2008 and C13* cells were exposed for 24 h to the same drug concentration (5 µM), close to the IC_50_ values of most Pd(phen) complexes. The Pd(phen)-thioureas actually accumulated from three to sevenfold more than the other complexes in both cell lines (Figure 3), almost reaching 800 pmol Pd/mg of protein, whereas Pt(bipy), Pd(bipy), and Pt(phen) complexes never reached 300 pmol Pt/mg or Pd/mg of protein. The Pd(phen) accumulation was always statistically larger than that of the other complexes with *p* < 0.001.

In trying to explain the differences in cellular accumulation as to why Pd(phen) accumulated at much higher levels, we evaluated the lipophilicity of our complexes. Although unable to distinguish between Pt or Pd complexes, the Chembiodraw ultra 12.0 software [16] gave us a useful hint to partially account for the greater accumulation of the Pd(phen) compounds, as it indicated a higher lipophylicity of phenanthroline complexes with respect to bipyridyl complexes, with logP values of 2.89 and 2.42, respectively. 

### 2.3. Pd(phen) Complexes Showed the Highest Affinity for DNA and Intercalation Ability

**Figure 4:**
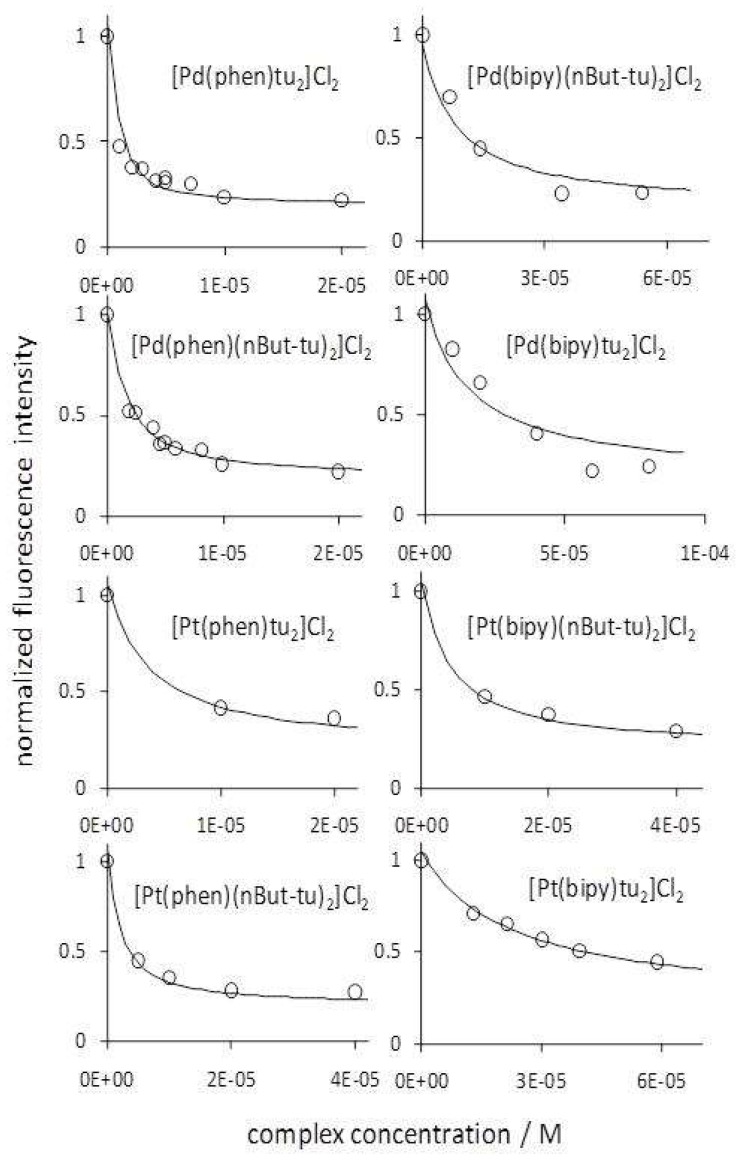
Fluorometric titrations for the displacement of ethidium bromide from calf-thymus DNA with Pt(II) and Pd(II) phen or bipy thiourea complexes. The estimated uncertainty of each data point was ± 10%. tu = thiourea; nBut = *n*-butyl. T = 22 ± 2 °C. Each curve represents the dependence of the normalized fluorescence intensity re-calculated as described in Section 4.5 of the Materials and Methods using the best dissociation equilibrium constant (K_d_) for each displacing metal complex.

In the next step of our effort to rationalize the cytotoxicity results, we tested the ability of these complexes to bind DNA by intercalating between bases [10,11,14,17]. We thus compared the intercalation ability of the eight complexes of the Series A and C by means of an ethidium bromide (EB) fluorometric displacement assay that took advantage of the much higher fluorescence quantum yield of DNA-bound EB relative to free EB [6,18]. The assay consisted in measuring the emission spectrum of EB in the presence of DNA while another DNA ligand able to displace EB was progressively added. To determine the DNA-binding affinity of the incoming ligand from analysis of the EB emission intensity values (see the Experimental Section 4.5 for the details), we need to know the DNA-binding properties, affinity, and stoichiometry, of the displaced ligand, EB in our case. From a Scatchard-type analysis (Figure S1), we determined the EB binding equilibrium constant and stoichiometry for the employed calf thymus DNA to be 1 × 10^6^ M^−1^ and 1 EB molecule per 2.5 base pairs, in keeping with reported values [19]. From the subsequent fluorometric titrations for the displacement of EB from DNA by a Pd or Pt complex (Figure 4), we determined the dissociation equilibrium constants, K_d_, and the corresponding ΔG° for the binding to DNA of the eight complexes investigated. These values are reported in Table 1 in order of decreasing binding affinity. It is quite apparent that phenanthroline complexes intercalated better than bipyridyl complexes. As for the metal, the Pd-phen combination performed only slightly better than the Pt-phen combination, whereas when bipy was the bidentate ligand no conclusion about the effect of a change in the metal could be drawn. The nature of the ancillary ligand seemed to affect affinity to some extent, though not in a consistent way in the complexes investigated. The introduction of the bulky *n*-butyl substituents on the thiourea ligands increased the affinity for DNA in three out of four tested complex pairs, namely Pt(phen) (ΔG° = 1.7 kJ mol^−1^), Pt(bipy) (ΔG° = 4.3 kJ mol^−1^), and Pd(bipy) (ΔG° = 1.7 kJ mol^−1^). On the other hand, a small affinity decrease (ΔG° = −1 kJ mol^−1^) was found with the Pd(phen) pair. 

### 2.4. Pd(phen) Complexes Caused the Greatest Perturbation of Cell Cycle and Apoptosis Induction

To assess whether a perturbation of the distribution of cells in the different phases of the cell cycle was a possible mechanism underlying the antiproliferative activity of these intercalating agents, the four Pd(bipy) and the four Pd(phen) complexes were tested in cell-cycle perturbation experiments on the 2008 and C13* cell lines, after 48 h of incubation at concentrations fixed approximately at the IC_50_ value of the most active Pd(phen) complexes. The percentage of cells in the different phases of the cell cycle is reported in Table 2. Untreated cells showed a normal diploid distribution presenting fast proliferation characteristics. Treatment with all eight compounds produced significant cell-cycle perturbation with higher accumulation of cells in the G_0_/G_1_ phase. The effect was dose-dependent, but to better compare the effect within each group of complexes, we show here the results obtained at 5 µM for all drugs. At this concentration, Pd(bipy) complexes caused only a slight perturbation of cell-cycle phase distribution and no apoptosis. However, at their IC_50_ concentration (approximately 50 µM), the effect was comparable with that of Pd(phen) drugs (data not shown). In our experimental conditions, 5 µM Pd(phen) greatly deranged the cell-cycle phase distribution of 2008 cells and, to an even greater extent, of the C13* cells. The marked accumulation in the G_0_/G_1_ cell-cycle phase was coupled to a decrease in the S and G_2_/M phases. Moreover, as a result of treatment with all Pd(phen) complexes, a significant increase of cell population in the sub-G_1_ phase was observed, which was indicative of apoptotic cell death (Table 2; Figure S2). In the resistant C13* cells, this effect was slightly reduced by the resistant phenotype. However, perturbation of cell-cycle and a great percentage of hypodiploid cells were still visible, in particular, after [Pd(phen)(nBu-tu)_2_]Cl_2_ and [Pd(phen)(Et_2_-tu)_2_]Cl_2_ treatment (Table 2; Figure S3).

### 2.5. Pd(phen) Complexes Targeted TOPO-II and Thymidylate Synthase (TS) Activity Affecting DNA Integrity

It is well known that intercalating agents, such as doxorubicin, can enter the nucleus and poison TOPO-II, also resulting in DNA damage and apoptotic cell death [20]. Because of this, we checked whether our DNA-intercalating metal complexes might differently affect the activity of this enzyme, thus accounting for the observed differential cytotoxicity.

**Figure 5:**
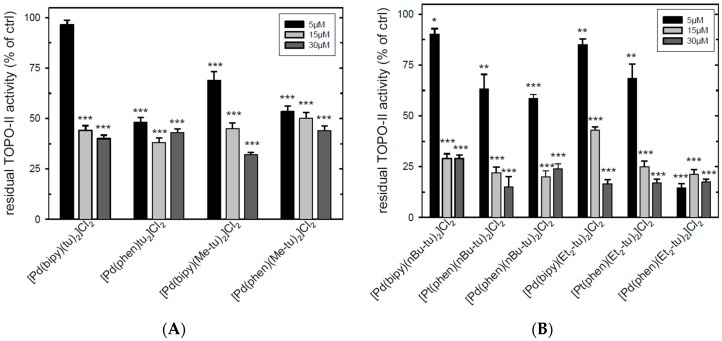
Inhibition of TOPO-II enzymatic activity. Inhibition of TOPO-II-dependent decatenation of kDNA following increasing concentrations of complexes versus dimethyl sulfoxide (DMSO) (arbitrarily set at 100%). (**A**) Effect of the indicated Pd(bipy) and Pd(phen) complexes of the A and B or (**B**) C and D series. The results represent the mean of three experiments conducted in duplicate. Error bars, SEM. * *p* < 0.05; ** *p* < 0.01; *** *p* < 0.001 versus control.

Figure 5 shows that most of the compounds were actually able to inhibit TOPO-II activity at the three tested concentrations. However, only phenantroline complexes, probably also correlating with the higher DNA binding affinity observed, reduced enzyme activity by at least 30% and 50% with Pt(phen) and Pd(phen) complexes, respectively, even at the lowest concentration (5 µM). A total of 80% inhibition was obtained with [Pd(phen)(Et_2_-tu)_2_]Cl_2_.

**Figure 6:**
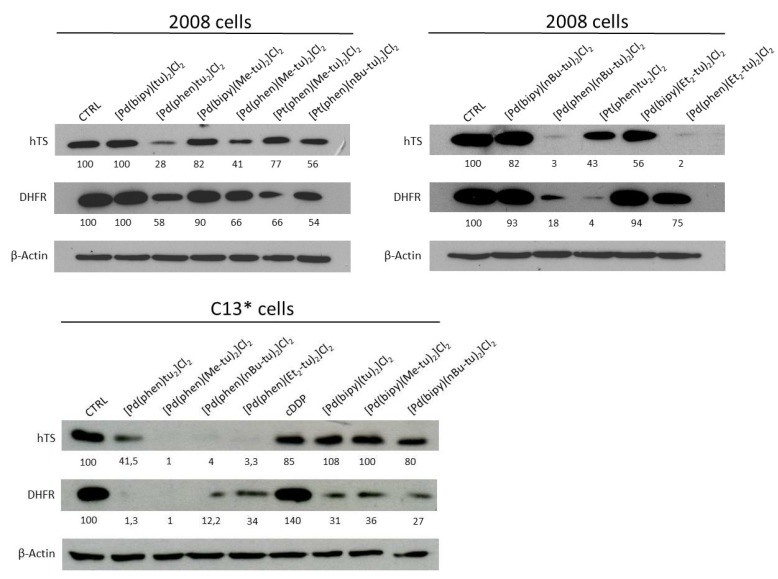
Effects of Pd(Pt)-bidentate ligand-thiourea complexes on thymidylate synthase (TS) and dihydrofolate reductase (DHFR) expression in 2008 and C13* cells. Western immunoblot analysis of TS and DHFR in cells treated for 24 h with the respective IC_50_ concentrations of the indicated complexes. human TS (hTS) monomer, molecular mass of 35 KDa, and DHFR monomer, molecular mass of 21 KDa. Representative blots of three independent experiments are shown. Human β-actin was used as internal control for protein loading. Numbers below the blots correspond to relative TS and DHFR quantification by densitometry compared with control (CTRL), arbitrarily set at 100.

Folate-cycle enzymes, thymidylate synthase (TS), and dihydrofolate reductase (DHFR) in particular can be included among the enzymes of DNA repair/substitution and cell cycle control, being essential for nucleotide synthesis. We thus hypothesized that a modulation of the expression of these enzymes by the here described DNA-intercalating metal complexes might contribute to the observed cytotoxicity. As shown in Figure 6, [Pd(phen)tu_2_]Cl_2_ actually reduced the TS and DHFR protein levels in 2008 cells by about 70% and 40%, respectively. Similarly, [Pd(phen)(Me-tu)_2_]Cl_2_ lowered the two protein levels by 60% and 35%. In these cells, [Pt(phen)(nBu-tu)_2_]Cl_2_ was also active in reducing the amount of both proteins by approximately 40%. The expression of TS was even more greatly affected by [Pd(phen)(nBu-tu)_2_]Cl_2_ and [Pd(phen)(Et_2_-tu)_2_]Cl_2_.

An interesting reduction of TS level was also observed with [Pt(phen)tu_2_]Cl_2_, and even more pronounced against DHFR. On the other hand, [Pd(bipy)(tu)_2_]Cl_2_, [Pd(bipy)(Me-tu)_2_]Cl_2_, and [Pd(bipy)(nBu-tu)_2_]Cl_2_, at their IC_50_ values slightly reduced TS level, but diminished DHFR protein level by over 60%. On the contrary, cisplatin was ineffective on the amount of DHFR but, as expected, reduced TS expression by about 20% [21,22]. In general, all (Phen)thioureas greatly inhibited the expression of TS and DHFR and, even if with some differences among the complexes, the reduction always exceeded 60% with respect to the control untreated samples.

**Figure 7:**
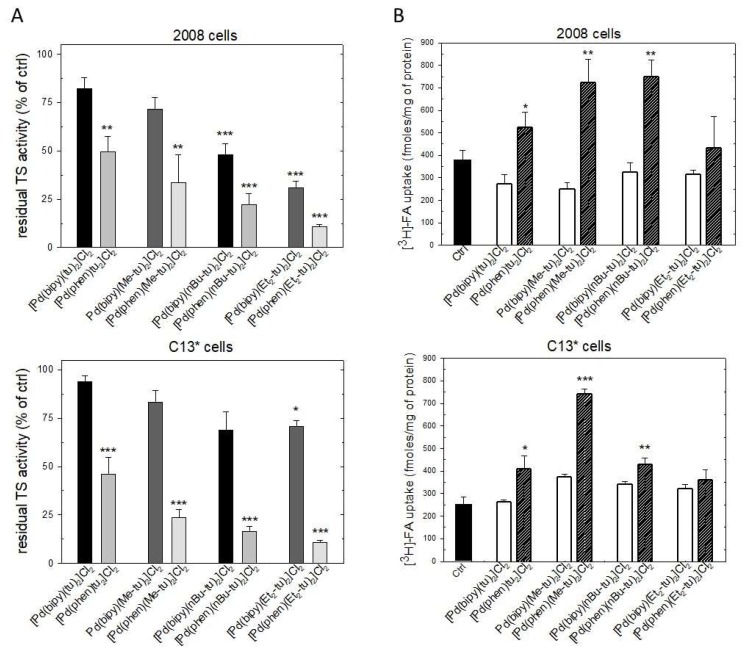
Effect of Pd(bipy)thiourea and Pd(phen)thiourea complexes on TS activity and folic acid (FA) uptake. (**A**) Residual TS activity of cellular extracts from 2008 and C13* cells processed after 48 h treatment with IC_50_ values of the complexes. (**B**) Effect of complexes on the uptake of [^3^H] folic acid in the 2008 cells and C13* cells. Error bars, SEM. * *p* < 0.05; ** *p* < 0.01; *** *p* < 0.001 versus control.

Together with the TS protein levels, the cellular TS activity was reduced by most of the tested complexes, particularly with 2008 cells. Again, Pd(phen) complexes showed the highest activity both on 2008 and C13* cells (Figure 7A). To the inhibition of TS protein expression, cells responded by increasing the uptake of folic acid (FA), probably as a result of a compensation mechanism, but unexpectedly this enhancement only followed treatment with Pd(phen)thioureas and not with the Pd(bipy) analogs, despite their effectiveness towards TS activity (Figure 7B).

## 3. Discussion

In this work, we analysed the effects of four series of bipyridyl (bipy) and phenanthrolyl (phen) complexes of Pt(II) or Pd(II) with thioureas in the same cell models previously exploited for evaluating the biological effects of six Pt(II)-bipy-thiourea complexes [6], four of which, namely, Pt(bipy)-A, -B, -C, and –D thiourea, were also included in the present study. Our goal here was to explore a broader variety of metal-bidentate ligand-thiourea complexes. We thus combined two metal ions with the same charge (2+) but different sizes, Pd(II) and Pt(II), with two bidentate ligands, bipy and phen, and four thioureas to see which ones among these 16 square planar metal complexes, previously synthetized and chemically characterized [10,11], featured the best biological and biochemical action against proliferation of human ovarian carcinoma cell lines (2008, C13*).

The biological data here obtained revealed that Pd(phen)-thioureas were the most cytotoxic complexes, with IC_50_ values lower by more than two orders of magnitude than the other complexes with the same thioureas, particularly with respect to Pt(bipy)-thiourea complexes. This difference in activity could partly result from a larger intracellular accumulation Pd(phen) complexes, suggesting that, regardless of the substituents on the thiourea moiety, combining Pd with the more lipophilic bidentate ligand, 1,10-phenanthroline, can allow a more efficient passive transport through the cell membrane [23] and/or a reduced efflux rate from cells. It is noteworthy in the fact that the Pd(phen) complexes of each series accumulated best also in the cDDP-resistant C13* cells, though in slightly smaller amounts than in the sensitive cells, suggesting a limited involvement of the resistance phenotype in their accumulation [24,25].

In addition to their greatest intracellular accumulation, Pd(phen) complexes confirmed their ability to intercalate DNA [26,27,28] and showed the highest affinities for the calf-thymus DNA base pairs among the tested complexes, including the corresponding bipy complexes. It has been reported that both AT and GC base pairs may stably bind the phenanthroline ligand, but its affinity is larger for the GC base pair [29]. In this regard, some Pd(phen) compounds, including our [Pd(phen)tu_2_]Cl_2_ and [Pd(phen)(Me-tu)_2_]Cl_2_, have been suggested to form hydrogen bonds with guanosine [14]. Therefore, our complexes bearing the phenanthroline moiety, in particular those with Pd(II), are likely powerful nucleic acid inhibitors. In particular, like the most clinically used DNA intercalating compounds [30], they may more strongly stabilize, lengthen, stiffen, and unwind the DNA double helix, thus concurring to the observed remarkable biological effect.

The physiological consequences of the direct alteration of DNA properties may add to the DNA damage originated from the interference of the metal complex with the activity of TOPO-II [31].

In this regard, we have previously shown that bipyridyl complexes of Pt(II) with thiourea are able to inhibit TOPO-II activity [9] and, more recently, Barra et al. showed that three Pd(phen) complexes, including our [Pd(phen)tu_2_]Cl_2_ and [Pd(phen)(Me-tu)_2_]Cl_2_, were also active as TOPO-II inhibitors in a dose-dependent manner [14]. We confirmed here that metal phenanthroline complexes were active TOPO-II inhibitors, and showed again that Pd(phen) were the most active TOPO-II inhibitors among the tested metal planar complexes, even at the smallest concentration tested. Although a mechanism of action cannot be exactly proposed [8,32], their interaction with DNA and TOPO-II may result in cell death via apoptosis [33].

A number of anticancer drugs acting merely via intercalation or alkylation of DNA, such as doxorubicin and cisplatin, cause DNA damage and perturbation of cell-cycle phase distribution, subsequently inducing apoptosis in cancerous cells [8,34].

In this work, the tested Pd(phen) thioureas were actually shown to cause a significant increase of cell population in the sub-G_1_ phase of 2008 and C13* cell lines, which was indicative of apoptotic cell death, and the greater arrest in the G_0_/G_1_ phase could be associated with TOPO-II inhibition and potential disturbances of replication or DNA damage. Arrest of cells in the G_1_ phase is thought to be an important cellular defence mechanism to prevent replication of damaged DNA [35].

Along with intercalation into DNA base pairs and binding to DNA-associated enzymes including TOPO-II [20], drugs such as doxorubicin are known to undergo other biological actions, ultimately contributing to DNA damage, with altered gene expression [34]. Candidate pharmacogenes for this action include the enzymes involved in the DNA repair mechanisms and the cell cycle control such as *TOP2A, MLH1, MSH2, TP53,* and *ERCC2* genes [36]. Two enzymes of the folate cycle, TS and DHFR, are also essential proteins for a normal cell cycle occurrence and DNA synthesis and ensure the correct substitution of damaged DNA after injury by chemotherapeutics [37]. In this work, the down-regulation of the TS protein expression by some complexes, especially Pd(phen), might reflect a characteristic similar to those observed with other DNA damaging agents [38]. The inhibitory effect of the complexes on TS can also result in imbalances of DNA precursors, such as dTMP, dTDP, and dTTP pools and others. In fact, the depletion of dTMP and subsequently dTTP induced perturbations in the levels of the other deoxynucleotides (dATP, dGTP, and dCTP), resulting in a reduction of the DNA synthesis and repair [39]. These effects suggest that a combinatorial strategy with our complexes and traditional TS inhibitors, such as 5-fluorouracil, raltitrexed or pemetrexed might have great potential in killing cells even with innated or more elevated TS levels that are more elevated [40].

Notably, the results shown here suggest that the cisplatin-resistance phenotype can only partly modulate these effects, as the complexes, Pd(phen) in particular, were almost equally effective against both sensitive and resistant cells, despite the over-expression, often observed in cDDP-resistant cells, of some of the enzymes involved in the pharmacology of these complexes, such as TOPO-II [41,42], TS, and DHFR [43], as well as the multidrug-resistant proteins [24,25].

In conclusion, we showed that among the 16 complexes obtained by combining the Pd(II) and Pt(II) metal ions, the bipy and phen bidentate ligands, and four differently substituted thioureas, that the Pd(phen)-thioureas are by far the most effective tumour-cell growth inhibitors. Their excellent cytotoxicities (low micromolar IC_50_s) likely stem from their remarkable intracellular accumulation, affinity for DNA-base pairs, and ability to modulate the expression of some key enzymes for DNA metabolism and cellular vitality, all properties in which they proved better than the other metal-bidentate ligand complexes tested (Pd(bipy), Pt(phen), and Pt(bipy), even in resistant cells.

Finally, the results also suggest that these active complexes may have potential for development as therapy for ovarian carcinoma based on drug combinations.

Nevertheless, additional pharmacological targets cannot be excluded, and more studies are necessary to deeply elucidate their mechanism of action.

## 4. Materials and Methods

### 4.1. Drugs and Chemicals

The synthesis of the bipy and phen complexes of Pt(II) or Pd(II) with the four thioureas and their chemical characterization by elemental analysis and ^1^H- and ^13^C[^1^H]-NMR spectroscopy have been reported previously [10,11]. All other chemicals were purchased from Sigma Chemical Co. (St. Louis, MO, USA), except where otherwise indicated.

### 4.2. Cell Lines

The human ovarian carcinoma cell lines 2008, A2780, and their respective resistant counterparts C13* and A2780/CP were used [44,45,46]. Cells were grown as monolayers in RPMI 1640 medium containing 10% heat-inactivated fetal bovine serum and 50 µg/mL gentamycin sulfate. All cell media and the serum were purchased from Lonza (Verviers, Belgium). Cultures were equilibrated with humidified 5% CO^2^ in air at 37 °C. All studies were performed in *Mycoplasma* negative cells, as routinely determined with the MycoAlert Mycoplasma detection kit (Lonza, Walkersville, MD, USA).

### 4.3. Cell Growth Assay

The inhibition of proliferation was measured by crystal violet staining, as previously reported [47], and the concentration at which cellular growth is inhibited by 50% (IC_50_) was determined following 48 h treatment with compounds. Briefly, the cell monolayer was fixed with methanol and stained with 0.05% crystal violet solution in 20% methanol for 1 h. The incorporated dye was solubilized in acidic isopropanol and determined spectrophotometrically with a multiplate reader (TecanGenios Pro with Magellan 6 software, San Diego, CA, USA) at 540 nm. The extracted dye was proportional to the cell number.

### 4.4. Intracellular Complex Accumulation

Intracellular metal concentrations in parts per billion were determined by inductively coupled plasma mass spectrometry (ICP-MS) [48,49] using a Plasma Spectrometer ICP-MS X Series^II^ (Thermo Fisher Scientific, Waltham, MA, USA) equipped with a CETAC ASX-520 autosampler (Neuss, Germany), an ablasion laser system NEW WAVE UP 213, a Scott double pass spray chamber, and a MicroMist nebulizer, at a sample uptake rate of approximately 0.25 mL min^−1^.

After treatment, the drug-containing medium was completely removed. Wells were washed thrice with PBS in order to make sure that each drug-exposed well was cleaned appropriately. Cell lysis was accomplished by addition of 0.5 mL of HCl 0.1 N and Triton X-100 0.1% for 1 h on the rocker. Subsequently, an aliquot of 400 μL was added to the same volume of sub-boiled HNO_3_ 69% and left at RT overnight to allow complete mineralization. On the next day, each sample was transferred into a 15 mL polypropylene tube and filled to a total volume of 8 mL with Milli-Q water. Finally, 8 mL of a Pt(II) or Pd(II) solution (final concentration 0.5 ppb) was added as the internal standard for the ICP-MS analysis, performed on the same day. The total amount of the respective metal per well was calculated and averaged over the three replicates. The Pt(II) and Pd(II) counts were normalized to the amount of cellular protein (nmol Pt or Pd/mg of protein).

### 4.5. Measurement of DNA-Intercalating Ability

The ability of each complex to intercalate DNA was determined by the ethidium bromide (EB) displacement fluorometric assay. Absorption measurements were performed on a double-beam Varian Cary100 UV-visible spectrophotometer with a cuvette containing the buffer on the reference beam. Fluorescence emission spectra were measured using a Horiba FluoroMax3 spectrofluorometer. The excitation wavelength was 525 nm and the emission spectra were measured between 540 and 720 nm. At such wavelengths, no interference on the EB excitation and emission came from the tested complexes. Also, 4 × 10 mm^2^ quartz cuvettes were always employed. For the experiment aimed at characterizing the binding affinity of EB to our calf thymus DNA, 100 µL of 20 µg/mL calf thymus DNA were added to 900 µL of HEN buffer and the emission spectrum was then measured at increasing concentrations of EB, determined spectrophotometrically using a molar extinction coefficient of 5680 M^−1^ cm^−1^ at 478 nm [50]. For the displacement experiments, 100 µL of a 20 µg/mL calf thymus DNA solution was added to 900 µL of HEN buffer and 20 µL of a 2.4 mM EB solution. The emission spectrum of EB was then measured at increasing concentrations of each added displacing compound until a stable emission spectrum, due to fully displaced EB, was obtained at 600 nm (I_600_). I_600_ was expressed as the sum of the contributions from free and DNA-bound EB. We first determined the instrumental proportionality constants between the concentration of each such form and the corresponding I_600_. Then, from the spectrophotometrically determined total DNA and EB concentrations, at each such concentration we calculated the fraction of bound base pairs with a 2.5:1 EB/base pair stoichiometry at saturation to obtain the EB/base pair association constant and the binding stoichiometry from Scatchard plot linear fitting, to determine the dissociation constants (K_d_) for the Pd and Pt complexes with the DNA, as previously developed [51,52]. In short, for each added metal complex, the measured I_600_ values were compared with the values calculated in terms of the previously determined EB/DNA binding constant and stoichiometry, an assumed K_d_ for the binding of DNA with the incoming metal complex, the stoichiometry being kept the same as found with EB, and the concentrations of EB, DNA base pairs, and metal complex. The best-fitting K_d_ value was kept. All experiments were performed at T = 22 ± 2 °C.

### 4.6. TOPO-II Decatenation Assay

TOPO-II activity was assayed in vitro through the Topoisomerase II assay kit (no. TG1001, TopoGEN Inc., Port Orange, FL, USA), following the instructions of the manufacturer. Nuclear extracts containing TOPO-II activity were obtained from cells and the ability to decatenate kDNA was analysed in the presence of DMSO and of the tested complexes, as previously shown [53]. Briefly, decatenation assay was performed with 50 ng kDNA in a 10 mL reaction mixture containing 50 mM Tris–HCl (pH 8.0); 150 mM NaCl; 10 mM MgCl_2_; 0.5 mM dithiothreitol; 2 mM ATP; 30 mg/mL BSA with DMSO; or 5, 15, 30 µM of compounds and 0.5 mg of cell nuclear extract. Reactions were incubated at 37 °C for 30 min and stopped by adding 5 mL of stop buffer (5% Sarkosyl 0.125% bromophenol blue and 25% glycerol). Samples were loaded directly onto a 1% agarose gel containing ethidium bromide (0.5 mg/mL). TOPO-II activity was measured by the appearance of decatenated minicircular products and determined as the percentage of DMSO by ImageJ software (Image J, USA National Institutes of Health, Bethesda, MD, USA).

### 4.7. Flow Cytometric Analysis of Cell Cycle

Quantitative measurements of the cell cycle phase distribution were performed by flow cytometry [54]. Cells were suspended in 0.5 mL of hypotonic fluorochrome solution (50 µg/mL PI, 0.1% sodium citrate, 0.1% Triton X-100). The samples were kept at 4 °C in the dark for at least 30 min, dispersed by repeated pipetting before flow cytometry analysis in a FACS Coulter Epics XL flow cytometer equipped with a single 488 nm argon laser. The percentage of nuclei in the different phases of the cell cycle (G0-G1, S, and G2-M) was calculated with a DNA cell cycle analysis software (Cell-Fit, Becton Dickinson). A minimum of 10^4^ cells/sample was analysed for each sample.

### 4.8. Western Immunoblotting

Enzymatic protein levels were evaluated as previously reported [47] by resolving 35 µg of each protein sample by SDS-PAGE (12%). The gels were electroblotted onto hydrophobic polyvinylidene difluoride membranes (Hybond-P PVDF, GE Healthcare Bio-Science, Uppsala, Sweden). Antibody staining was performed with a chemiluminescence detection system (ECL Plus Western Blotting Detection Reagent, GE Healthcare Bio-Science, Uppsala, Sweden) using a 1:500 dilution of the mouse anti-human TS (TS106) monoclonal primary antibody (Invitrogen S.r.L., Milan, Italy), 1:1000 dilutions of the mouse anti-human DHFR monoclonal antibody (Tebu-Bio, Milan, Italy), and 1:1000 of mouse anti-human ß-actin antibody (Sigma-Aldrich S.r.L., Milan, Italy) in conjunction with a 1:3000 dilution of horseradish peroxidase-conjugated sheep anti-mouse secondary antibody (GE Healthcare Bio-Science, Uppsala, Sweden). Quantification of signal intensity was performed by densitometry on a GS-800 calibrated densitometer (Bio-Rad) and analysed by using Quantity One software (Bio-Rad, Hercules, CA, USA).

### 4.9. TS Catalytic Assay

Pellets from exponentially growing cells were thawed by the addition of ice-cold lysis buffer (200 mM Tris-HCl pH 7.4, 20 mM 2-mercaptoethanol, 100 mM NaF, and 1% Triton X-100), sonicated (three times 5 s with intervals of 5 s), and centrifuged at 14,000× *g* for 15 min at 4 °C. The supernatant was used for TS catalytic assay [43] that was based on the measurements of the amounts of ^3^H release from 5-[^3^H]dUMP during its TS catalysed conversion to dTMP. Briefly, the reaction was started by adding 5-[^3^H]dUMP (1 µM final concentration, specific activity 5 mCi/mol) to enzyme suspensions in assay buffer and 650 µM 5,10-methylenetetrahydrofolate in a final volume of 50 µL. After incubation for 60 min at 37 °C and blocking by adding 50 µL of ice-cold 35% trichloroacetic acid, residual 5-[^3^H]dUMP was removed by the addition of 250 µL of 10% neutral activated charcoal. The charcoal was removed by centrifugation at 14,000× *g* for 15 min at 4 °C, and a 150 µL sample of the supernatant was assayed for tritium radioactivity in the liquid scintillator analyzer Tri-Carb 2100 (Packard Inc., Conroe, TX, USA).

### 4.10. Radioligand Assay for Folic Acid

To assess folic acid accumulation, uptake studies were conducted with 30 nM [^3^H] folic acid (FA) (specific activity 0.5 Ci/mmol) at 37 °C [55] in the presence or absence of the tested compounds. Then, 24 h after seeding, the culture medium was replaced with folate-free RPMI 1640 medium for at least an additional 24 h to maximize the externalization of folate receptor to cell surface. After washing, 0.1 N NaOH and 0.1% Triton X-100, and 550 μL of this cell lysate were analysed for radioactivity using a scintillation counter (Tri-Carb 2100, Packard Inc., Conroe, TX, USA) to relate ³H-FA accumulation to cellular protein content. 

### 4.11. Statistical Analyses

All values report the mean ± the standard error of the mean (SEM), unless otherwise indicated. *p*-values were calculated with two-sided Student’s *t*-test and ANOVA followed by the Tukey′s multiple comparison. * *p* < 0.05; ** *p* <0.01; *** *p* < 0.001. 

## Figures and Tables

**Table 1:** Dissociation equilibrium constants and corresponding ΔG° for the binding of the given metal complexes to calf thymus DNA.

Complex	K_d_/10^−8^ M	ΔG°/kJ mol^−1^
[Pd(phen)tu_2_]Cl_2_	6	40.8
[Pd(phen)(nBut-tu)_2_]Cl_2_	9	39.8
[Pt(phen)(nBut-tu)_2_]Cl_2_	20	37.8
[Pt(phen)tu_2_]Cl_2_	40	36.1
[Pt(bipy)(nBut-tu)_2_]Cl_2_	50	35.6
[Pd(bipy)(nBut-tu)_2_]Cl_2_	100	33.9
[Pd(bipy)tu_2_]Cl_2_	200	32.2
[Pt(bipy)tu_2_]Cl_2_	280	31.3

The uncertainty on the equilibrium constants (K_d_) values is estimated to ± 30%. tu = thiourea, nBut = *n*-butyl. T = 22 ± 2 °C.

**Table 2:** The distribution (%) of 2008 and C13* cells in the different phases of cell cycle after treatment with the Pd-bipy-thiourea and Pd-phen-thiourea complexes.

Drugs (5 µM)	2008 Cells				C13* Cells			
	G_0/_G_1_	S	G_2_/M	Apoptosis	G_0/_G_1_	S	G_2_/M	Apoptosis
Control	66.9 ± 3	11.5 ± 1	14.6 ± 3.5	1.53 ± 1	62.7 ± 4	17.1 ± 2	13.6 ± 2	1.4 ± 0.2
[Pd(bipy)(tu)_2_]Cl_2_	69.7 ± 3	12.1 ± 1	14.1 ± 0.2	1.24 ± 0.5	59.7 ± 4	19.8 ± 1	13.7 ± 1	2.1 ± 0.3
[Pd(bipy)(Me-tu)_2_]Cl_2_	63.5 ± 5	20.5 ± 3	11.8 ± 1	1.44 ± 0.4	54.2 ± 2	22.7 ± 4	16.8 ± 2	3.4 ± 1.5
[Pd(bipy)(nBu-tu)_2_]Cl_2_	73.5 ± 6	12.6 ± 3	12.8 ± 4	1.12 ± 0.2	67.8 ± 5	13.9 ± 2	15.3 ± 3	1.32 ± 0.4
[Pd(bipy)(Et_2_-tu)_2_]Cl_2_	69.6 ± 5	12.7 ± 2	14.2 ± 3	1.30 ± 0.3	66.4 ± 7	14.9 ± 2	14.4 ± 3	1.37 ± 0.4
[Pd(phen)tu_2_]Cl_2_	77.2 ± 2	3.2 ± 0.8	4.1 ± 0.2	9.8 ± 1.7	81.6 ± 2	2.3 ± 0.3	3.1 ± 0.3	10.6 ± 0.5
[Pd(phen)(Me-tu)_2_]Cl_2_	71.8 ± 1	3.6 ± 1.4	4.6 ± 1.8	14.8 ± 0.7	81.1 ± 2	2.4 ± 0.5	3.4 ± 1.2	9.5 ± 2
[Pd(phen)(nBu-tu)_2_]Cl_2_	64.7 ± 2	2.2 ± 0.1	7.2 ± 2.2	24.5 ± 1.1	71.1 ± 6	12.7 ± 2	5.1 ± 2	11.6 ± 2
[Pd(phen)(Et_2_-tu)_2_]Cl_2_	73.3 ± 3	2.4 ± 0.9	5.6 ± 0.8	16.8 ± 0.6	78.6 ± 4	4.5 ± 0.6	3.6 ± 0.4	12.7 ± 3

24 h after seeding, the cells were exposed to 5 µM of each drug for 48 h, then DNA content of untreated and treated cells was determined by flow cytometry after propidium iodide staining. All results shown are representative of two/three independent assays.

## Supplementary Materials

Supplementary materials can be found at https://www.mdpi.com/1422-0067/20/24/6122/s1.

## Abbreviations

cDDP	Cisplatin or *cis*-diamminedichloridoplatinum(II)
Phen	Phenanthroline
Bipy	Bipyridine
Tu	Thiourea
TOPO-II	Topoisomerase-II
TS	Thymidylate synthase
DHFR	Dihydrofolate reductase
ICP-MS	Inductively-coupled plasma mass spectrometry
EB	Ethidium bromide
FA	Folic acid
P-gp	P-glycoprotein
