# Supplementary material for: The 1,10-Phenanthroline Ligand Enhances the Antiproliferative Activity of DNA-Intercalating Thiourea-Pd(II) and -Pt(II) Complexes Against Cisplatin-Sensitive and -Resistant Human Ovarian Cancer Cell Lines
Source: Int J Mol Sci. 2019 Dec 4;20(24):6122. doi: 10.3390/ijms20246122 (PMC6969938; doi:10.3390/ijms20246122)
Supplement: Supplementary file 1 [file ijms-20-06122-s001.pdf]

The 1,10-Phenanthroline Ligand Enhances the Antiproliferative Activity of DNA-Intercalating thiourea-Pd(II) and -Pt(II) Complexes Against Cisplatin-Sensitive and -Resistant Human Ovarian Cancer Cell Lines

Gaetano Marverti [1,*], Gaia Gozzi [1], Angela Lauriola [1,3], Glauco Ponterini [2], Silvia Belluti [2], Carol Imbriano [2], Maria Paola Costi [2] and Domenico D'Arca [1,*]

[1] Department of Biomedical, Metabolic and Neural Sciences, Via Campi 287, University of Modena and Reggio Emilia, Modena 41125, Italy; gaetano.marverti@unimore.it (G.M.); g.gozzi@holostem.it (G.G.); angela.lauriola@univr.it (A.L.); domenico.darca@unimore.it (D.D)

[2] Department of Life Sciences, Via Campi 213/d, University of Modena and Reggio Emilia, Modena 41125, Italy; glauco.ponterini@unimore.it (G.P.); silvia.belluti@unimore.it (S.B.); carol.imbriano@unimore.it (C.I.); mariapaola.costi@unimore.it (MP.C.)

[3] Current address: Department of Biotechnology, University of Verona, Verona 37134, Italy

* Correspondence: gaetano.marverti@unimore.it (G.M.); domenico.darca@unimore.it (D.D).

Received: 15 November 2019; Accepted: 2 December 2019; Published: December 2019

Supplementary Information

Table 1. IC_{50} values (μM ±SD) for the complexes against four human cancer cell lines.

			**2008 cells**				
**Pt(bipy)A**<br>[Pt(bipy)tu$_2$]Cl$_2$	240.6±32	**Pt(phen)1**<br>[Pt(phen)tu$_2$]Cl$_2$	>100	**Pd(bipy)H**<br>[Pd(bipy)(tu)$_2$]Cl$_2$	50±6	**Pd(phen)A**<br>[Pd(phen)tu$_2$]Cl$_2$	4.5±0.5
**Pt(bipy)B**<br>[Pt(bipy)(Me-tu)$_2$]Cl$_2$	145.4±18	**Pt(phen)6**<br>[Pt(phen)(Me-tu)$_2$]Cl$_2$	59.4± 6	**Pd(bipy)I**<br>[Pd(bipy)(Me-tu)$_2$]Cl$_2$	37.7±4	**Pd(phen)B**<br>[Pd(phen)(Me-tu)$_2$]Cl$_2$	7.2±0.3
**Pt(bipy)C**<br>[Pt(bipy)(nBu-tu)$_2$]Cl$_2$	137.3±16	**Pt(phen)7**<br>[Pt(phen)(nBu-tu)$_2$]Cl$_2$	75.5± 6	**Pd(bipy)L**<br>[Pd(bipy)(nBu-tu)$_2$]Cl$_2$	51.3±7	**Pd(phen)C**<br>[Pd(phen)(nBu-tu)$_2$]Cl$_2$	3.9±0.2
**Pt(bipy)D**<br>[Pt(bipy)(Et$_2$-tu)$_2$]Cl$_2$	142.4±15	**Pt(phen)2**<br>[Pt(phen)(Et$_2$-tu)$_2$]Cl$_2$	41.5 ± 5	**Pd(bipy)-M**<br>[Pd(bipy)(Et$_2$-tu)$_2$]Cl$_2$	55.3±4	**Pd(phen)-D**<br>[Pd(phen)(Et$_2$-tu)$_2$]Cl$_2$	2±0.2
			**C13* cells**				
**Pt(bipy)A**<br>[Pt(bipy)tu$_2$]Cl$_2$	445.7±51	**Pt(phen)1**<br>[Pt(phen)tu$_2$]Cl$_2$	>100	**Pd(bipy)H**<br>[Pd(bipy)(tu)$_2$]Cl$_2$	55±7	**Pd(phen)A**<br>[Pd(phen)tu$_2$]Cl$_2$	8.8±0.5
**Pt(bipy)B**<br>[Pt(bipy)(Me-tu)$_2$]Cl$_2$	312.3±29	**Pt(phen)6**<br>[Pt(phen)(Me-tu)$_2$]Cl$_2$	71.7±6	**Pd(bipy)I**<br>[Pd(bipy)(Me-tu)$_2$]Cl$_2$	65.6±8	**Pd(phen)B**<br>[Pd(phen)(Me-tu)$_2$]Cl$_2$	4.1±0.2
**Pt(bipy)C**<br>[Pt(bipy)(nBu-tu)$_2$]Cl$_2$	155.5±12	**Pt(phen)7**<br>[Pt(phen)(nBu-tu)$_2$]Cl$_2$	87.5±10	**Pd(bipy)L**<br>[Pd(bipy)(nBu-tu)$_2$]Cl$_2$	45.6±5	**Pd(phen)C**<br>[Pd(phen)(nBu-tu)$_2$]Cl$_2$	4.8±0.5
**Pt(bipy)D**<br>[Pt(bipy)(Et$_2$-tu)$_2$]Cl$_2$	161.3±13	**Pt(phen)2**<br>[Pt(phen)(Et$_2$-tu)$_2$]Cl$_2$	38.1±4	**Pd(bipy)-M**<br>[Pd(bipy)(Et$_2$-tu)$_2$]Cl$_2$	50.3±6	**Pd(phen)-D**<br>[Pd(phen)(Et$_2$-tu)$_2$]Cl$_2$	3.4±0.5
			**A2780 cells**				
**Pt(bipy)A**<br>[Pt(bipy)tu$_2$]Cl$_2$	224.8±23	**Pt(phen)1**<br>[Pt(phen)tu$_2$]Cl$_2$	108.8±13	**Pd(bipy)H**<br>[Pd(bipy)(tu)$_2$]Cl$_2$	60±7	**Pd(phen)A**<br>[Pd(Fen)tu$_2$]Cl$_2$	23±4
**Pt(bipy)B**	125.4±15	**Pt(phen)6**	51.4±4	**Pd(bipy)I**	52.3±6	**Pd(phen)B**	18.1±3

[Pt(bipy)(Me-tu)$_2$]Cl$_2$		[Pt(phen)(Me-tu)$_2$]Cl$_2$		[Pd(bipy)(Me-tu)$_2$]Cl$_2$		[Pd(phen)(Me-tu)$_2$]Cl$_2$	
**Pt(bipy)C** [Pt(bipy)(nBu-tu)$_2$]Cl$_2$	123.7±13	**Pt(phen)7** [Pt(phen)(nBu-tu)$_2$]Cl$_2$	63.3±7	**Pd(bipy)L** [Pd(bipy)(nBu-tu)$_2$]Cl$_2$	30.5±7	**Pd(phen)C** [Pd(phen)(nBu-tu)$_2$]Cl$_2$	8.5±2
**Pt(bipy)D** [Pt(bipy)(Et$_2$-tu)$_2$]Cl$_2$	134.1± 21	**Pt(phen)2** [Pt(phen)(Et$_2$-tu)$_2$]Cl$_2$	41.5±5	**Pd(bipy)-M** [Pd(bipy)(Et$_2$-tu)$_2$]Cl$_2$	34.8±4	**Pd(phen)-D** [Pd(phen)(Et$_2$-tu)$_2$]Cl$_2$	10±2
			**A2780/CP cells**				
**Pt(bipy)A** [Pt(bipy)tu$_2$]Cl$_2$	405.4±35	**Pt(phen)1** [Pt(phen)tu$_2$]Cl$_2$	>100	**Pd(bipy)H** [Pd(bipy)(tu)$_2$]Cl$_2$	53.3±6	**Pd(phen)A** [Pd(Fen)tu$_2$]Cl$_2$	19.5±3
**Pt(bipy)B** [Pt(bipy)(Me-tu)$_2$]Cl$_2$	342.6±32	**Pt(phen)6** [Pt(phen)(Me-tu)$_2$]Cl$_2$	66.3±5	**Pd(bipy)I** [Pd(bipy)(Me-tu)$_2$]Cl$_2$	45.9±6	**Pd(phen)B** [Pd(phen)(Me-tu)$_2$]Cl$_2$	15.3±3
**Pt(bipy)C** [Pt(bipy)(nBu-tu)$_2$]Cl$_2$	185.7±21	**Pt(phen)7** [Pt(phen)(nBu-tu)$_2$]Cl$_2$	67.2±4	**Pd(bipy)L** [Pd(bipy)(nBu-tu)$_2$]Cl$_2$	34.5±5	**Pd(phen)C** [Pd(phen)(nBu-tu)$_2$]Cl$_2$	13.7±2
**Pt(bipy)D** [Pt(bipy)(Et$_2$-tu)$_2$]Cl$_2$	151.2±23	**Pt(phen)2** [Pt(phen)(Et$_2$-tu)$_2$]Cl$_2$	43.1±6	**Pd(bipy)-M** [Pd(bipy)(Et$_2$-tu)$_2$]Cl$_2$	38±5	**Pd(phen)-D** [Pd(phen)(Et$_2$-tu)$_2$]Cl$_2$	9.1±3

The IC_{50} is defined as the concentration causing 50% growth inhibition in treated cells when compared to control cells after 48 h drug exposure. Values are means ± SEM of three separate experiments performed in duplicate.

Table S2. IC_{50} values and resistant factors (RF) for Pd(phen)-thioureas, cisplatin and doxorubicin against the 2008 and C13* cell lines. (IC_{50} (μM) ± SD and resistant factor, RF=IC_{50} resistant/IC_{50} parent line).

Complexes	2008 cells	C13* cells	RF
[Pd(phen)tu$_2$]Cl$_2$	4.5±0.5	8.8±0.5	1.95
[Pd(phen)(Me-tu)$_2$]Cl$_2$	7.2±0.3	4.1±0.2	0.57
[Pd(phen)(nBu-tu)$_2$]Cl$_2$	3.9±0.2	4.8±0.5	1.23
[Pd(phen)(Et$_2$-tu)$_2$]Cl$_2$	2.0±0.2	3.4±0.5	1.70
Cisplatin	1.55 ± 0.13	11.3 ± 0.42	7.30
Doxorubicin	0.054 ± 0.007	0.157 ± 0.016	2.91

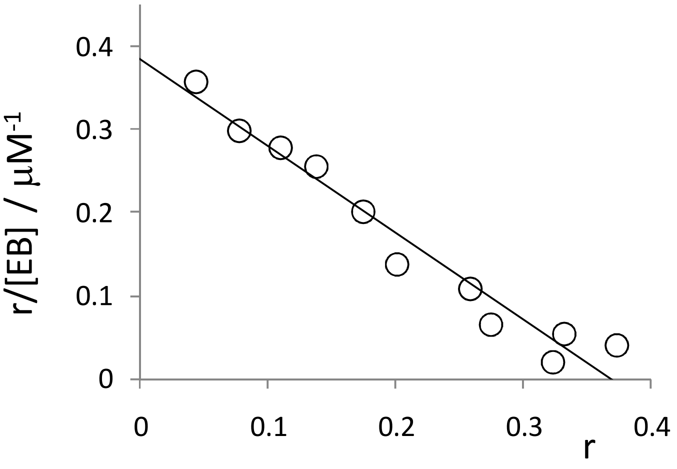

Figure S1. Scatchard plot for the ethydium bromide (EB)/calf thymus DNA binding equilibrium. r = fraction of occupied EB-binding sites in the DNA; [EB] = molar concentration of free EB. T = 22 ± 2 °C.

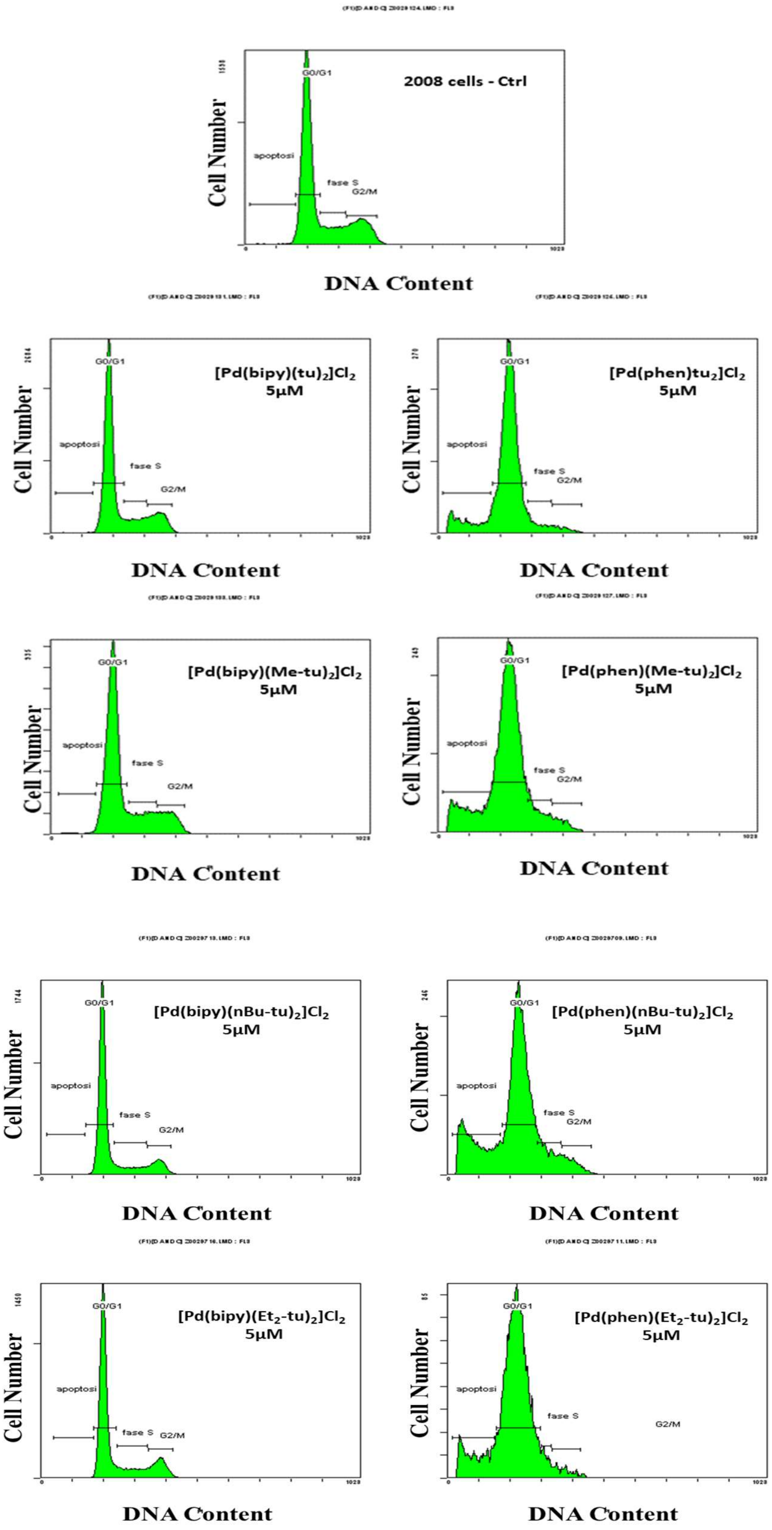

Figure S2. Cell cycle-related analysis of the cDDP-sensitive human cancer 2008 cells after treatment with the Pd(bipy)-thiourea and Pd(phen)-thiourea complexes. Cells were seeded in 6-well plates for 24 h, and then treated with the indicated drug concentration. After 48 hr, cells were harvested and subjected to cell cycle analyses as described in Methods. All results shown are representative of two/three independent assays.

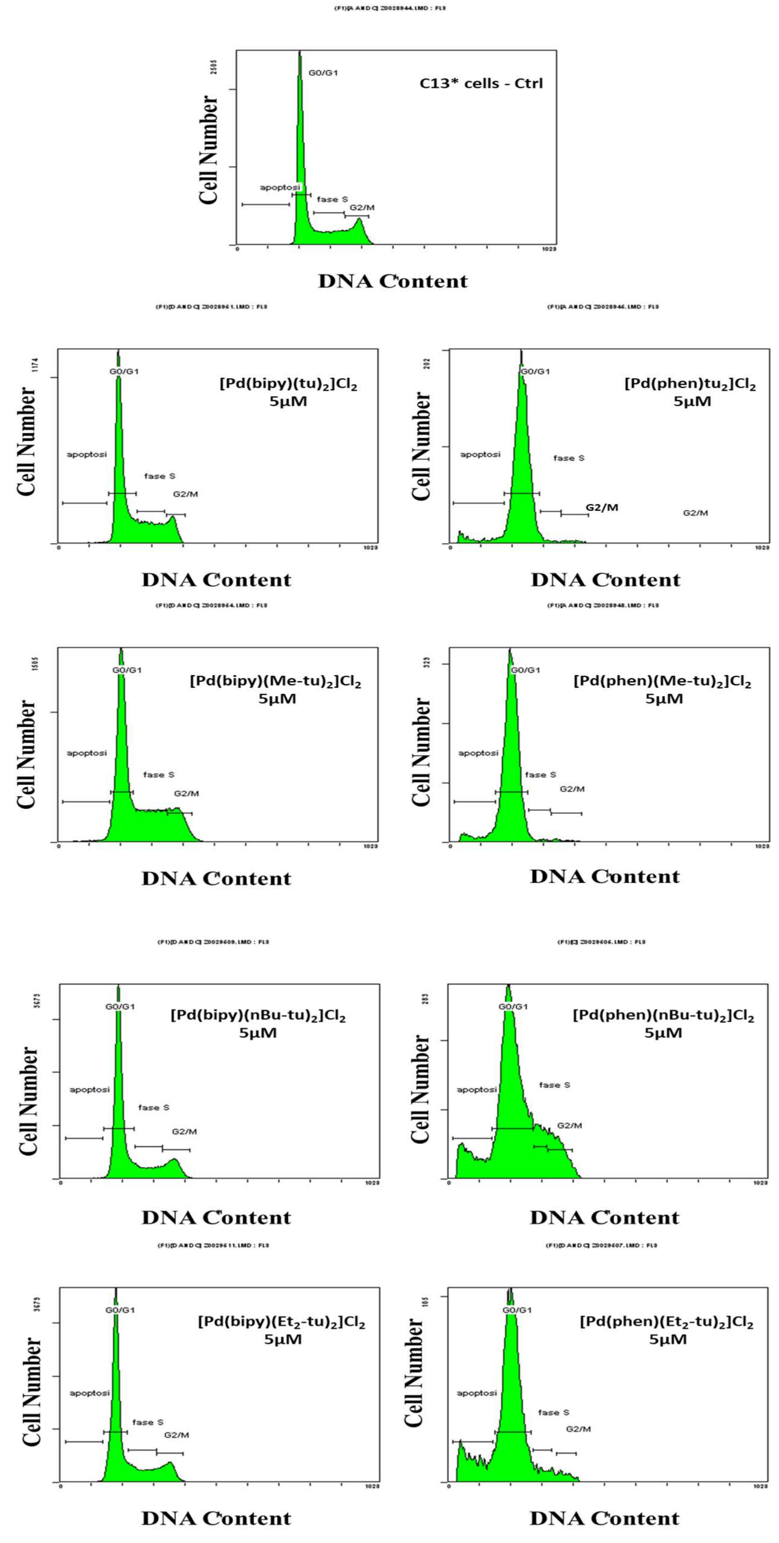

Figure S3. Cell cycle-related analysis of the cDDP-resistant human cancer C13* cells after treatment with the Pd(bipy)-thiourea and Pd(phen)-thiourea complexes. Cells were seeded in 6-well plates for 24 h, and then treated with the indicated drug concentration. After 48 hr, cells were harvested and subjected to cell cycle analyses as described in Methods. All results shown are representative of two/three independent assays.
